# Hepatoid adenocarcinoma of the lung: Presenting mediastinal metastasis without transfer to the liver

**DOI:** 10.3892/ol.2014.2064

**Published:** 2014-04-15

**Authors:** YI-QUN CHE, SHUANG WANG, YANG LUO, JING-BO WANG, LU-HUA WANG

**Affiliations:** 1 Clinical Laboratory, Cancer Institute and Hospital, Chinese Academy of Medical Science and Peking Union Medical College, Beijing 100021, P.R. China; 2 Department of Radiology, Cancer Institute and Hospital, Chinese Academy of Medical Science and Peking Union Medical College, Beijing 100021, P.R. China; 3 Department of Medical Oncology, Cancer Institute and Hospital, Chinese Academy of Medical Science and Peking Union Medical College, Beijing 100021, P.R. China; 4 Department of Radiation Oncology, Cancer Institute and Hospital, Chinese Academy of Medical Science and Peking Union Medical College, Beijing 100021, P.R. China

**Keywords:** hepatoid adenocarcinoma, lung, α-fetoprotein, immunohistochemistry, metastasis, chemoradiation

## Abstract

Hepatoid adenocarcinoma of the lung (HAL) is a rare type of lung cancer. Its diagnosis and treatment may be difficult due to the varied presentation; however, immunohistochemical analysis facilitates the diagnosis. The present study presents a case of HAL. The patient was a 48-year-old male who presented with a primary complaint of back pain. A chest computed-tomography scan revealed a lobulated soft-tissue mass that extended from the left lung apex to the middle and posterior mediastinum. The area of the largest cross-section was 7.9×10.0 cm and the lymph nodes did not demonstrate metastasis. Immunohistochemical staining of a transbronchial lung biopsy revealed that the tumor cells were α-fetoprotein (AFP)(positive) and hepatocytes(positive) and a diagnosis of hepatoid carcinoma of the left lung was established. The level of serum AFP, a tumor marker, was elevated (6,283 ng/ml). The patient presented with mediastinal metastases and was classified as stage IIIA (N2); following diagnosis, the patient received concurrent chemoradiation. Subsequent to chemoradiation, the left lung lump with the largest cross-section was 3.3×4.2 cm and the serum AFP had fallen to its lowest level (23.11 ng/ml). However, when the patient relapsed, the serum AFP level elevated markedly (57,800 ng/ml). Furthermore, the nodules of metastasis increased in number and enlarged, with the largest measuring 2.1 cm. The patient succumbed as a result of a lung infection.

## Introduction

Hepatoid adenocarcinoma (HAC) is a rare form of adenocarcinoma that is defined by morphological and functional hepatic differentiation (1). To the best of our knowledge, few cases have been reported in the literature that have shown the prognosis of this subtype of lung adenocarcinoma (Table I). The present study reports an extremely rare case of HAC of the lung (HAL) presenting with α-fetoprotein (AFP) production.

During embryonic development, the lung, liver and stomach are derived from the primitive foregut. Due to certain abnormalities in differentiation, the adenocarcinoma cells from specific organs, including the lungs, differentiate into liver cells. Studies have shown that this type of tumor is able to generate products, including albumin, α-antitrypsin, prothrombin, ferritin, transferrin and AFP, which are usually produced by normal liver cells and liver cancer cells. Therefore, this type of tumor is termed a HAC. The majority of HACs originate from the stomach and HACs that originate from the lungs are extremely rare. A study by Ishikura *et al* (2) defined these lung cancers as lung liver adenocarcinoma. This type of cancer exhibits a liver cell-like differentiation and morphology, and is AFP(positive) in histopathological analysis.

The present study includes immunohistochemical analyses, serum AFP levels, chest computed tomography (CT) images, the clinical course of poor prognosis through concurrent chemoradiation and adjuvant chemotherapy, and a relatively long-term follow-up. The study was approved by the ethics committee of the Cancer Hospital, Chinese Academy of Medical Sciences, National GCP Center for Anticancer Drugs (Beijing, China). The patient provided written informed consent.

## Case report

**Figure 1:**
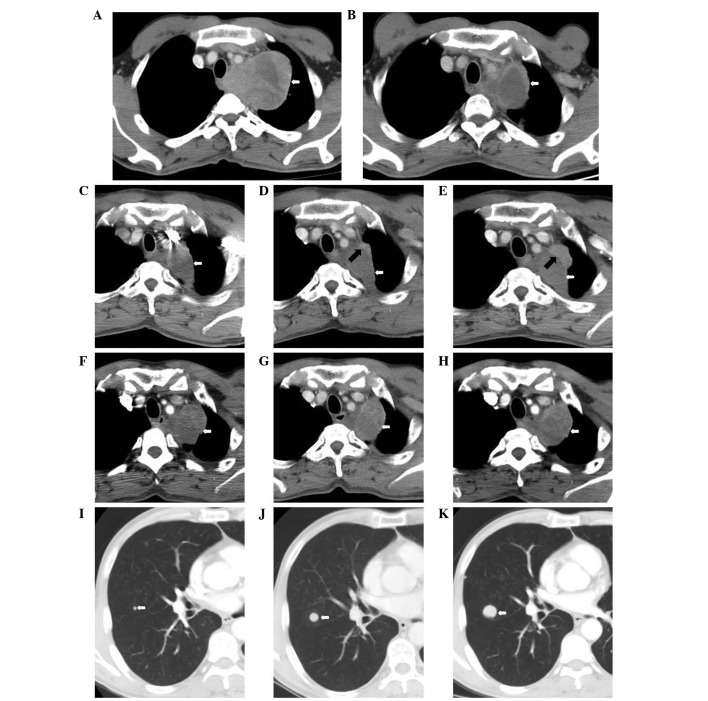
Chest computed tomography (CT) during the clinical course. (A) Pre-treatment on July 29, 2011 of the left lung apex to the middle and posterior mediastinum. The area of the largest cross-section is 7.9×10.0 cm (white arrow). (B) Post-concurrent chemoradiation on September 16, 2011. CT of the soft-tissue mass shows a maximal cross-sectional area of 5.3×4.6 cm (white arrow). (C) November 22, 2011, the left lung apex to the middle and posterior mediastinum following four cycles of paclitaxel + cisplatin chemotherapy and concurrent chemoradiation. The area of the largest cross-section is 3.3×4.2 cm (white arrow). (D) March 7, 2012, pulmonary primary lesions of the anterior and lateral to the adjacent irregular nodules following five cycles of docetaxel + nedaplatin chemotherapy. The area of the largest cross-section is 0.7×2.1 cm (black arrow). (E) May 14, 2012, pulmonary primary lesions of the anterior and lateral to the adjacent irregular nodules. The area of the largest cross-section is 1.6×3.1 cm (black arrow). (F) July 26, 2012 and (G) September 13, 2012, the left lung lump of the largest cross-section is 4.9×4.8 cm (white arrow). (H) October 25, 2012, the left lung lump of the largest cross-section is 5.0×4.0 cm (white arrow). (I) July 26, 2012, the nodes in the image demonstrate nodules of metastatic carcinoma and the largest is ~0.4 cm. (J) September 13, 2012 and (K) October 25, 2012, the nodules of metastasis increased and enlarged. The largest were ~1.2 and ~2.1 cm, respectively.

**Figure 2:**
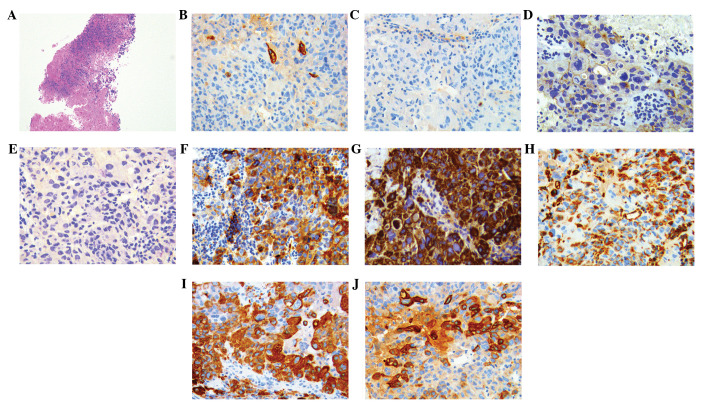
Histological findings of tumor and coloration of the immunohistochemistry. (A) Tumor cells are arranged with cancer nests and adenoids (hematoxylin and eosin staining; original magnification, ×100). Coloration of the immunohistochemistry: (B) Cytokeratin (CK)7 (positive); (C) CK20(negative), (D) renal cell carcinoma(negative), (E) thyroid transcription factor-1(negative); (F) α-fetoprotein(positive); (G) hepatocyte(positive); (H) vimentin(positive); (I) CK18(positive); and (J) pan-cytokeratin [AE1/AE3(positive)] (SP staining; original magnification, ×400).

**Figure 3:**
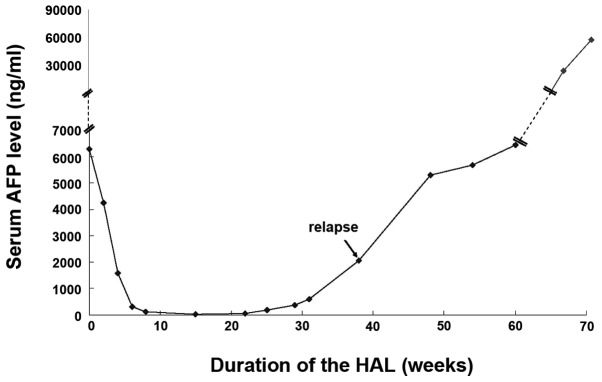
Serum AFP level for the duration of the HAL. AFP, α-fetoprotein; HAL, hepatoid adenocarcinoma of the lung.

A 48-year-old Chinese male was admitted to the Cancer Institute and Hospital, Chinese Academy of Medical Sciences and Peking Union Medical College (Beijing, China) due to the primary complaint of back pain over a 6-month period, which became progressively worse in the latter 3 months. The patient was 174-cm tall and weighed 65 kg, resulting in a body surface area of 1.81 m^2^. The patient had a 70 pack-year smoking history of 30 years, however, did not report a dry or productive cough and experienced no palpitations or chest tightness. The patient did not have alcoholic hepatitis or a relevant family history. The patient’s Karnofsky performance status (KPS) score was 90. There was no abnormality in the abdomen and the pretreatment AFP levels progressively increased from 1,926 to 6,283 ng/ml. A chest-CT scan demonstrated a lobulated soft-tissue mass, extending from the left lung apex to the middle and posterior mediastinum, and the longest cross-sectional dimension of the mass was 7.9×10.0 cm (Fig. 1A). An abdominal-CT scan manifested a 1.5×1.5 cm contrast-enhancing nodule on the posterior lobe of the liver, which was diagnosed as a hemangioma by the radiologist. No enlarged lymph nodes were found on the retroperitoneal and bilateral inguinal areas. A pretreatment ultrasound examination revealed that no clear nodules or masses were observed elsewhere in the liver, gallbladder, spleen or pancreas. No enlarged lymph nodes were found in the abdominal cavity, retroperitoneal or inguinal area. A bronchoscopy demonstrated a bloody discharge in the subsegmental bronchus in the apex of the posterior segment of the upper lobe of the left lung. Brain magnetic resonance imaging showed that the bilateral ventricles were symmetrical and no obvious enlargement or shift was observed. There were no abnormal enhanced nodules or masses in the bilateral cerebral hemispheres, cerebellum or pons. Complete blood counts, urinalysis, coagulation studies and chemical analyses were normal. Except for the elevation of AFP serum levels, the tumor markers, including carcinoembryonic antigen, carbohydrate antigen 125, squamous cell carcinoma antigen, neuron-specific enolase and cytokeratin (CK) 19 fragment, were within the normal ranges. Hepatitis B virus (Hb) surface antigen, Hb surface antibody, hepatitis C virus, treponema pallidum and human immunodeficiency virus were all negative. The pathological morphology of the biopsy from the mediastinal lymph node supported the diagnosis of lung cancer. Immunohistochemical analyses demonstrated positive staining for CK7 in a marginal number of cells, and AFP(positive), pan-cytokeratin [AE1/AE3(positive)], CK18(positive), vimentin(positive), hepatocyte(positive), CK20(negative), renal cell carcinoma(negative) and thyroid transcription factor-1(negative) (Fig. 2), indicating mediastinal lymph node involvement of HAL. The patient received concurrent chemoradiation between August 2, 2011 and September 13, 2011. Radiation therapy of 60 Gy/2 Gy/30 F was delivered through intensity-modulated radiation therapy using 6-MV X-rays, concomitant with two cycles of paclitaxel plus cisplatin. A CT scan demonstrated a partial response following the concurrent chemoradiation, with a maximal cross-sectional area of 5.3×4.6 cm and the hilar lymph was ~0.5×0.9 cm (Fig. 1B). Subsequent to four cycles of paclitaxel plus cisplatin chemotherapy and concurrent chemoradiation, the area of the largest cross-section of the left lung apex to the middle and posterior mediastinum was 3.3×4.2 cm (Fig. 1C). There was a decrease in the AFP serum levels to 23.11 ng/ml observed in the period between October 22, 2011 and March 6, 2012 (Fig. 3). Subsequently, the patient continued to receive consolidative chemotherapy. However, following five cycles of consolidative chemotherapy, the AFP serum level increased to 386.8 ng/ml again and correspondingly the CT scan showed new pulmonary lesions (Fig. 1D), indicating disease progression. Therefore, the patient received a different chemotherapy regimen (docetaxel and nedaplatin) and the serum level of AFP increased to 2,070 ng/ml two months after five cycles of the regimen (Fig. 3). A repeated CT scan did not demonstrate metastasis in the liver, however, the size of the lung lesions continued to increase (Fig. 1E), an additional imaging examination showed tumor recurrence. The patient received regular follow-up (Table II). The left lung lump of the largest cross-section was 5.0×4.0 cm, and the nodules of metastasis increased and enlarged, with the largest being ~2.1 cm (Fig. 1F–H). On March 17, 2013, the patient succumbed to a lung infection.

## Discussion

Stomach HAC is the most common AFP-producing carcinoma, accounting for ~2.5–15% of gastric cancers (3–5). However, AFP-producing lung cancer is rarely reported, and its pathological cell features and clinical symptoms remain unclear. Thus far, no standard treatment strategy is available for lung-originated HAC. The patient profile in the current study is of a middle-aged male and heavy smoker with the primary clinical symptom of back pain. In addition, the serum AFP level of the patient was significantly elevated. The diagnosis of HAL was primarily based on morphological features and immunohistochemical markers facilitated the diagnosis. Mediastinal metastasis of this disease has not yet been reported. According to the literature, the combined treatment of surgical resection and adjuvant tegafur-uracil chemotherapy is a common selection for HAL treatment (6), and surgery was not considered in the present study. To prevent the continuing enlargement of the tumor from compressing the respiratory tracts and esophagus, radiotherapy to the mediastinum and concurrent chemotherapy were applied to control the disease. Radiotherapy was considered to be an appropriate treatment, as the patient was in a condition of physical tolerance, with a KPS score of 90 and was able to tolerate radiotherapy. Additionally adenocarcinoma is relatively sensitive to irradiation. However, radiotherapy has several disadvantages; it is a type of local treatment approach and metastasis may occur during the treatment. The relapse in this patient was detected via observation of an increase in serum AFP level, and this was identified significantly earlier than any indication from imaging findings.

Serum AFP was clearly increased in the patient of the present study and was also detected in the cytoplasm by immunohistochemical staining. However, AFP is not unique to HAL and is more commonly found in hepatocellular carcinoma, cholangiocarcinoma and teratomatous germ cell tumors (7). Therefore, other immunohistochemical stains are necessary to confirm the diagnosis of HAL. Monoclonal antibody hepatocyte expression appears to be confined to normal and neoplastic liver cells and sensitivity for this is heightened compared with AFP. Hepatocyte reactivity has been shown regularly in hepatocellular carcinomas, hepatoblastomas and HAC. However reactivity in cholangiocarcinomas and metastatic tumors to the liver is rare. Immunostaining for CKs is helpful in defining HAL. CK18, marker of simple parenchyma, was positive in HAL, while CK20 was negative in HAL; staining for CK7 can be positive (8) or negative (9).

Since HAL is an extremely heterogeneous type of tumor, there is currently no standard treatment. HAL is generally treated as an adenocarcinoma of the common type, derived from the involved organ system. Patients with localized tumors undergo surgery (10) and those with mediastinal metastases are treated with concurrent chemoradiation as opposed to surgery. The patient in the present study obtained an initial partial response following four cycles of paclitaxel plus cisplatin chemotherapy and concurrent chemoradiation. The patient subsequently underwent five cycles of docetaxel plus nedaplatin chemotherapy, until the disease progressed. Transbronchial needle aspiration tissues were not enough to detect the molecular markers of therapeutic significance, including epidermal growth factor receptor and anaplastic lymphoma kinase mutations.

Another indication for the poor prognosis of HAL is the production of AFP, which possesses immunosuppressive properties (11). The poor prognosis was considered to be associated with the extensive venous invasion and locally advanced or metastatic presentation (12). The unfavorable outcome of the patient in the present study may be due to a combination of various factors, including late unresectable disease and a short progression-free survival time following chemotherapy.

The diagnosis of HAL in the patient was confirmed and the focus of the present study is the clinical significance of the initial elevated serum AFP level. One possible reason for this is that there were two primary tumors, lung cancer and liver cancer. The timely diagnosis and treatment of the lung lesions avoided the misdiagnosis of lung cancer. However, as this patient had no previous history of liver disease and did not exhibit abnormal liver function, it was unlikely to conclude that the AFP abnormality was associated with liver disease. It was more likely that the disease was associated with lung cancer and the multiple lesions in the liver were pulmonary metastases. This type of lung cancer with elevated serum AFP levels is a special variety. It is noteworthy that certain lung cancers possess similar morphological features to hepatocellular carcinoma or liver cell-like differentiation. Immunohistochemical detection on a mediastinal needle-biopsy specimen further confirmed the diagnosis, thereby saving valuable time that could be used for treatment.

By identifying and exploring the aforementioned circumstances, clinicians should be aware that in addition to liver cancer, other malignancies, including lung cancer, may be accompanied by elevated serum AFP levels. Comprehensive systemic examinations and combined diagnostic examinations are, therefore, required to prevent misdiagnosis.

In conclusion, the heterogeneity of HAC complicates the diagnosis. Furthermore, the associated poor prognosis emphasizes the requirement for an accurate and early diagnosis using immunohistochemistry as an increase in serum AFP levels occurs significantly earlier compared with any indications from imaging examinations. However, the optimal management of HAL is still not well defined and requires further investigation.

## Figures and Tables

**Table I:** Clinical pathological data of cases of hepatoid adenocarcinoma of the lung reported in the literature.

							Prior to treatment					
										
First author, Year (ref)	Gender	Age, years	Diagnosis	Stage	Location	Size, cm	Metastases	Serum AFP, ng/ml	Tissue AFP	Therapy	Time for AFP levels to return to normal	Prognosis
Yoshino I, 1996 (13)	M	54	ADSCC	I	LU	2.0	No	696		Surgery	2 weeks post surgery	24 months, A
Arnould L, 1997 (14)	M	36	SCC	-	LU	10.0	No	11,600	+	Surgery	19 weeks post surgery	7 months, S
Nasu, 1998 (15)	M	63	LCNEC	-	RU	8.0	No	14,000	+	-	-	11 months, S
Hayashi Y, 2002 (16)	M	55	AD	IB	RU	6.0	No	/	+	Surgery	Postoperative	32 months, A
Hiroshima K, 2002 (17)	M	71	SCC	IIIA	RL	10.5	No	7,417	+	Surgery + radiotherapy	>2 year	>2 years, S
	M	70	SCC	IIIB	RU	6.0	No	24.3	/	Surgery	-	>2 years, S
	F	64	LCNEC	IIIB	RL	-	No	74.4	/	Chemotherapy + surgery	-	>2 years, S
Kitada *et al*, 2011 (6)	M	69	AD	IIIA	RL	7.0	Lymph node	4,620	+	Surgery	2 weeks post surgery	A

M, male; F, Female; ADSCC, adenosquamous cell carcinoma; SCC, squamous cell carcinoma; LCNEC, large cell neuroendocrine carcinoma; AD, adenocarcinoma; LU, left upper lobe; RU, right upper lobe; RL, right lower lobe; AFP, α-fetoprotein; S, succumbed; A, alive; +, positive; /, not tested.

**Table II:** CT/MRI and tumor marker examinations of the patient’s clinical course.

Date	Clinical course	AFP-ng/ml (07)^a^	CEA ng/ml (0–5)^a^	CA125 U/ml (0–35)^a^	SCC ng/ml (0–1.5)^a^	Cyfra21-1 ng/ml (0–3.3)^a^	NSE ng/ml (0–18)^a^	LDH U/l (135–225)^a^	CT/MRI
July 29, 2011	Prior to treatment	6,283	4.16	8.05	0.5	2.33	14.34	327	The left lung apex to the middle and posterior mediastinum, the area of the largest cross-section was 7.8×7.9×10.0 cm
August 15, 2011	Concurrent	4,241	4.11	8.54	0.5	4.25	12.71	346	-
August 30, 2011	Chemoradiation	1,581	3.44	8.90	0.5	4.08	11.25	182	-
September 16, 2011	Following concurrent chemoradiation	318.7	3.05	7.19	0.6	2.44	10.58	145	CT of the soft tissue mass showed a maximum cross sectional area of 5.3×4.6 cm and the size of hilar lymph nodes were reduced to ~0.5×0.9 cm
September 29, 2011		118.5	3.78	10.26	0.7	2.06	15.33	202	-
October 12, 2011		-	2.96	-	-	-	-	191	Brain MRI showed no abnormal enhanced nodules
November 22, 2011	Following 4 cycles of paclitaxel + cisplatin	23.11	3.10	13.69	1.0	2.97	14.71	159	The left lung apex to the middle and posterior mediastinum, the area of the largest cross-section was 3.3×4.2 cm
December 6, 2011	Chemotherapy								
January 17, 2012		70.33	3.69	9.83	0.7	2.02	10.06	186	-
February 6, 2012	Following 4 cycles of docetaxel + nedaplatin chemotherapy	193.6	4.24	8.02	0.5	1.66	9.84	129	-
March 7, 2012	Following 5 cycles of docetaxel + nedaplatin chemotherapy	386.8	3.18	7.54	0.5	1.35	9.03	141	Pulmonary primary lesions of the anterior and lateral to the adjacent irregular nodules, the area of the largest cross section was 0.7×2.1 cm
March 19, 2012		588.2	3.37	7.94	0.8	1.48	9.49	119	-
May 14, 2012	Follow-up	2,070	-	-	-	-	-	-	Pulmonary primary lesions of the anterior and lateral to the adjacent irregular nodules, the area of the largest cross section was 1.6×3.1 cm
August 1, 2012	Following 3 cycles of cytoxan + epirubicin + cisplatin chemotherapy	5,303	-	-	-		-	-	The left lung lump with the largest cross section was 4.9×4.8 cm, the large node of metastatic carcinoma was ~0.4 cm
September 14, 2012	Following 1 cycle of gemcitabine + carboplatin	5,682	4.01	11.24	0.6	1.92	11.43	269	The nodules of metastasis increased in number by five and enlarged, the largest was ~1.2 cm
October 25, 2012	Follow up	6,438	-	-	-	-	-	-	The left lung lump with the largest cross section was 5.0×4.0 cm, the largest nodule was ~2.1 cm
December 12, 2012	Icaritin	24,989	-	-	-	-	-	-	-
January 10, 2013		57,800	-	-	-	-	-	-	-

a Normal range.

CT, computed tomography; MRI, magnetic resonance imaging; AFP, α-fetoprotein; CEA, carcinoembryonic antigen; CA125, carbohydrate antigen 125; SCC, squamous cell carcinoma antigen; cyfra21-1, cytokeratin 19 fragment; NSE, neuron-specific enolase; LDH, lactate dehydrogenase; -, not tested.
